# Antiquated ejection fraction: Basic research applications for speckle tracking echocardiography

**DOI:** 10.3389/fphys.2022.969314

**Published:** 2022-10-24

**Authors:** Sarah L. Sturgill, Vikram Shettigar, Mark T. Ziolo

**Affiliations:** Frick Center for Heart Failure and Arrhythmia, Department of Physiology and Cell Biology, Davis Heart and Lung Research Institute, The Ohio State University, Columbus, OH, United States

**Keywords:** cardiovascular performance, heart performance, contractility, echocardiography, speckle tracking

## Abstract

For years, ejection fraction has been an essentially ubiquitous measurement for assessing the cardiovascular function of animal models in research labs. Despite technological advances, it remains the top choice among research labs for reporting heart function to this day, and is often overstated in applications. This unfortunately may lead to misinterpretation of data. Clinical approaches have now surpassed research methods, allowing for deeper analysis of the tiers of cardiovascular performance (cardiovascular performance, heart performance, systolic and diastolic function, and contractility). Analysis of each tier is crucial for understanding heart performance, mechanism of action, and disease diagnosis, classification, and progression. This review will elucidate the differences between the tiers of cardiovascular function and discuss the benefits of measuring each tier *via* speckle tracking echocardiography for basic scientists.

## 1 Introduction

Proper heart function is mandatory for the quintessential operation of the cardiovascular system. Thus, for proper evaluation of cardiovascular performance, all constituents of heart performance (systolic function, diastolic function, and contractility) must be assessed. An increase in metabolic demand from the body (in situations such as exercise) mandates an increase in cardiac output to meet this demand. The heart increases cardiac output *in vivo via* three mechanisms: by the Bowditch effect (i.e., increasing heart rate), Starling’s Law of the heart (i.e., increasing end diastolic volume), and through sympathetic (i.e., β-adrenergic) stimulation (Klabunde, 2012). These influences on the heart utilize the cardiac reserve, augmenting systolic and diastolic function. Contractility is a key contributor to systolic function and cardiac reserve. Unfortunately, cardiovascular disease is the leading cause of death in the world and proper evaluation of all aspects of heart performance is crucial in determining the development and progression of heart disease (Tan, 1986). Thus, the *in vivo* assessment of all tiers of cardiovascular performance, including left ventricular systolic and diastolic function and contractility, is significant to understand how well the heart is performing and its role in the development and progression of heart disease.

### 1.1 The tiers of cardiovascular performance

The overall health of cardiovascular performance (definition can be found in Table 1) can be deduced by ejection fraction (EF) and mean arterial pressure (MAP) (Eqs 1, 2). Indices of cardiovascular performance are highly dependent upon preload, afterload, and heart rate. Preload is measured by End Diastolic Volume (EDV) and afterload, or the systemic pressure to overcome for the heart to eject blood, is correlated to MAP. A major contributor to poor cardiovascular performance is heart disease (e.g, heart failure, HF).

**TABLE 1:** Definitions and measurements of the different tiers.

Tier	Definition	Parameter	Methodology
Cardiovascular performance	Determined by how well the pulmonary and peripheral vasculature and the heart are functioning	Mean arterial pressure	Tail cuff, catheter, telemetry
		Ejection fraction	M-mode, speckle tracking
Heart performance	Determined by how the atria and ventricles are functioning, and is determined by systolic and diastolic function	Cardiac output	M-mode, speckle tracking
		Stroke volume	M-mode, speckle tracking
Systolic function	Occurs when the ventricle is contracting, and is determined by heart rate, preload, afterload and contractility	Fractional shortening	M-mode, speckle tracking
		dP/dt_max_	Intra-ventricular catheterization
Diastolic Function	Occurs when the ventricle is relaxed and filling with blood, and is determined by preload, heart rate and ventricular compliance	Mitral valve filling	Speckle tracking, power doppler, tissue doppler
		Diastolic strain rate	Speckle tracking
		dP/dt_min_	Intra-ventricular catheterization
Contractility	Innate ability of the heart to eject a SV at a given preload/afterload	dP/dt/EDV	Intra-ventricular catheterization
		Strain	Speckle tracking
		Strain rate	Speckle tracking

The performance of the heart is complex and can be broken down into systolic and diastolic function and contractility. Heart performance is a subset of cardiovascular performance, and thus is dependent upon preload, afterload, heart rate, and contractility. Heart performance (definition can be found in Table 1) is measured by cardiac output (CO), which takes into account the electrical and mechanical function, or by stroke volume, which takes into account only the mechanical function (Eqs 3, 4).

Systolic function (definition can be found in Table 1) can be measured by fractional shortening, which is dependent upon preload, afterload, heart rate, and contractility (Eqs 5, 6). Diastolic function (definition can be found in Table 1) can be measured by E/A ratio (measured by Doppler ultrasound) or E’/A’ ratio (measured by left ventricular wall velocity), which measures the ratio of early to late ventricular filling *via* mitral valve flow. E/A ratio is dependent upon preload, ventricular compliance, and heart rate.

The definition of contractility is heavily debated. The definition can be as complicated as “the preload, afterload and length-independent intrinsic kinetically controlled, chemo-mechanical processes responsible for the development of force and velocity” (Muir and Hamlin, 2020). For this discussion, contractility is considered an intrinsic property of the cardiomyocyte and is inherently preload-, afterload-, and heart rate-independent. Hence, contractility contributes to systolic function, which, along with diastolic function, contribute to heart performance, which in turn contributes to cardiovascular performance.

**FIGURE 1:**
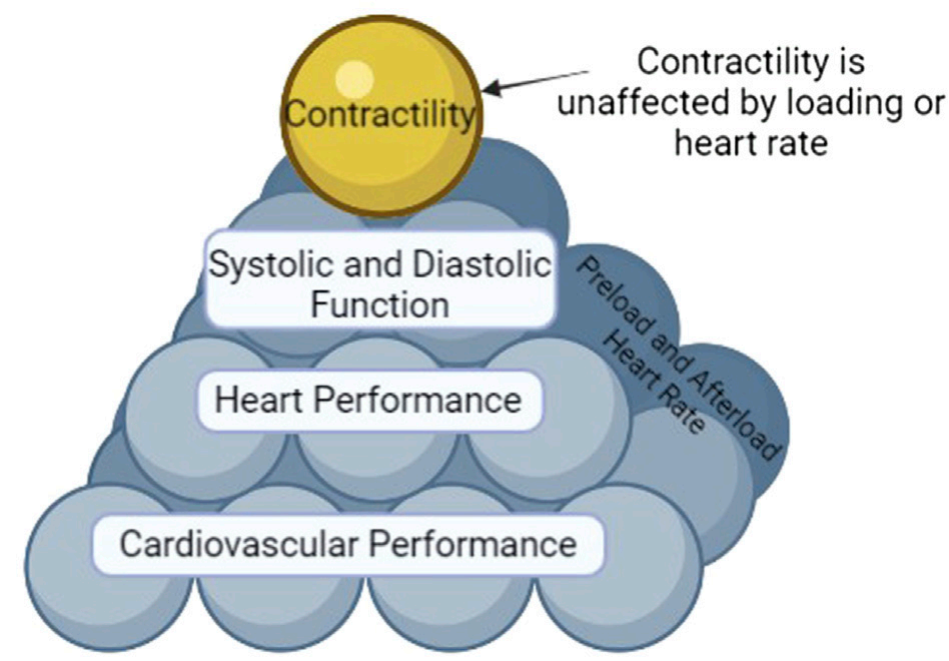
The Pyramid of Cardiovascular Performance. Affecting all tiers except for contractility are heart rate and loading parameters (preload and afterload). Cardiovascular performance encompasses the function of all tiers and is thus the bottom of the pyramid. Heart performance is a component of cardiovascular performance. Systolic and diastolic function and contractility are components of heart performance. However, AS contractility is by definition an inherent, load and heart rate independent characteristic of the myocyte, it sits at the top of the pyramid, unaffected by heart rate or load like the other tiers.

Shown in Figure 1 are the different tiers (or factors) that determine cardiovascular performance. Contractility is on top of the pyramid since it is an independent variable (not affected by load and heart rate independent) (Muir and Hamlin, 2020). Contractility contributes to systolic function. Systolic and diastolic function (both of which are load and heart rate dependent) contribute to heart performance. Heart performance contributes to cardiovascular performance. Proper functioning of each tier is necessary for health. Hence, as each tier is interconnected, to understand cardiovascular performance and to be able to truly understand mechanisms, classifications, and prognosis of disease it is vital that one measures *in vivo* systolic and diastolic function and contractility and not just cardiovascular performance (i.e., EF).

#### 1.1.1 Tiers of cardiovascular performance in the clinics

While there are multiple means to diagnose heart disease in patients (physical exam, blood tests, noninvasive such as cardiac imaging (echocardiography, CT scan, MRI, etc.), stress test, electrocardiogram, and invasive such as angiography and cardiac catheterization), a common choice for diagnosis is echocardiography due to cost and ease. Typically, a special emphasis is placed on EF, which can be calculated through a multitude of techniques ranging in accuracy.

EF can be measured by short or long-axis M-mode, which follows the displacement of the walls of the heart along a user-drawn line, which has user-dependent error and is highly variable. The most accurate method of measuring EF is 3D echocardiography to measure full LV chamber volume at systole and diastole (Lang et al., 2015). Alternatively, the most accurate 2D method to calculate EF is Modified Simpson’s method, also known as the Biplane of Discs model recommended by the American Society of Echocardiography (Lang et al., 2015). While this method does allow for an accurate calculation of EF compared to traditional M-mode measurements, it is rarely applied in basic research despite increased accuracy. While EF varies in accuracy of quantification, it still is dependent on loading parameters and heart rate.

EF is antiquated and often misstated as an assessment of systolic function or contractility. This is incorrect as EF is highly preload- and heart rate-dependent and thus cannot be a measure of contractility (Kass et al., 1987). *In vivo* modulators of heart function (Bowditch, Starling’s Law, and sympathetic) result in changes to preload, a greater SV, and thus increased CO (Ricci et al., 1979; Sequeira and van der Velden, 2015). These resultant changes in ventricular volume are associated with corresponding changes in EF, since these volumes are used in the calculation of EF (please see Section 5- Equations). For example, with Starling’s Law, this is usually associated with a decrease in EF (due to increased EDV even though SV increases), while sympathetic stimulation will increase EF (Mangano et al., 1980; Stratton et al., 1987). Also, changes in EDV (i.e., Starling’s Law) will result in changes in left ventricular end diastolic dimension and thus, fractional shortening (FS). There is also a misconception in the field that EF equates to systolic function. For example, a high EF is not necessarily indicative of healthy or superior heart function and must be interpreted in conjunction with SV. A high EF but with low SV (due to low EDV) likely suggests a hypertrophic heart. Preservation or improvement of EF is a common occurrence in models of concentric hypertrophy, and often presents with comorbidities such as hypertension, obesity, diabetes, renal dysfunction, etc (Hieda et al., 2020; Mouton et al., 2020). Conversely, a low EF is not necessarily indicative of worsened heart function, as the heart can remodel both pathologically and physiologically to increase preload. Physiological remodeling through exercise can cause decreased baseline EF, but increased cardiac reserve (Roof et al., 2013). EF is also highly afterload dependent (Kolh et al., 2003). Thus, there may be changes in EF that may not be due to altered heart performance (i.e., hypertension). Further, a normal EF does not preclude changes to heart performance, as there may be corresponding changes to volumes. This can clearly be observed in HF, a syndrome in which the heart cannot pump enough blood to meet the body’s requirements. There are two classifications of HF: reduced (HFrEF) or preserved EF (HFpEF). Since EF has been presumed to be a measure of systolic function and/or contractility, HFrEF is also known as systolic HF, while HFpEF is also known as diastolic HF. However, there is both systolic and diastolic dysfunction in both types of HF (Daubert Melissa, 2019; Pfeffer et al., 2019). Thus, cardiologists realized that a better understanding of heart performance (ventricular systolic function, diastolic function, and contractility) was needed for better diagnosis and treatment for cardiac patients as EF is insufficient.

Clinicians have implemented quantitative methods that assess all tiers of cardiovascular function since the early 2000s (Konstam and Abboud, 2017). Since its clinical implementation, speckle tracking echocardiography (STE) has been used on a day-to-day basis, resulting in earlier diagnosis and therefore better treatment options for patients (Pérez et al., 1992; Inaba et al., 2005; Ciarka et al., 2021; Pastore et al., 2021). Clinicians have established proper assessment of the tiers of cardiovascular function in determining their treatment of human patients. This has ultimately improved the understanding of cardiovascular health and treatment.

#### 1.1.2 Tiers of cardiovascular performance in research

Researchers commonly perform standard methods of echocardiography (M-mode measurements) and with good reason. This method remains the simplest and cheapest method to assess cardiovascular function over multiple time points throughout an experiment, allowing for assessment of progression of disease. However, this method falls short of assessing all tiers of cardiovascular performance. Specifically, standard echocardiography cannot assess diastolic function or cardiac contractility. Researchers often revert to alternative methods of assessing cardiovascular function, such as intra-left ventricular catheterization for pressure-volume analysis (PV loops) (Peterson et al., 2018). Although this method does provide indices for all tiers of cardiovascular performance (cardiovascular performance, heart performance, systolic and diastolic function, and contractility) with the added benefit of decreased load dependence, it is unfortunately a terminal procedure.

Contractility is a difficult, yet necessary index to measure in research settings. There is an abundance of wide-ranging reasons that scientists need to properly measure contractility. Clinicians have learned that in order to determine how the heart is impacted through various treatments, proper characterization of the cardiovascular system at all tiers is essential. Inherently, contractility is an intrinsic property of the myocyte. Excitation-contraction coupling (ECC) is the process by which the cardiac myocyte contracts and changes in myocyte contraction will alter contractility (Bers, 2002). Hence to examine the *in vivo* effects of alterations to ECC proteins (Wang et al., 2012; Nixon et al., 2013; Nixon et al., 2014), signaling pathways targeting ECC proteins (Traynham et al., 2012), etc. as potential therapeutic strategies for heart disease, one should measure contractility to correctly understand the resultant effects on systolic function, diastolic function, heart performance, and cardiovascular performance. Measuring *in vivo* contractility (and the effects of Bowditch, Starling’s law, and sympathetic) is also important in helping to ascertain disease mechanisms, testing if new treatment strategies (i.e., drugs, devices, regenerative medicine, etc.) will be beneficial, etc. Another traditional *in vivo* measurement of contractility is performed *via* intra-left ventricular catheterization to measure pressure-volume changes in the heart. Indices such as dP/dt_max_ and dP/dt_min_ (normalized to volume) reflect isovolumetric contraction and relaxation and can be obtained from PV loops (Rhodes et al., 1997). Unfortunately, as previously mentioned, this is a terminal procedure.

Although this review is focused on contractility, with the recent explosion of HFpEF, it is also vitally important to measure diastolic function. Unfortunately M-mode echocardiography, unlike STE, does not provide any measures of diastolic function.

## 2 Use and interpretation of parameters of cardiovascular function in research

Due to the small heart size and fast heart rate, there are a lack of techniques to measure true parameters of heart performance in mice. Thus researchers, all too frequently, misuse parameters of cardiovascular performance, heart performance, systolic function, diastolic function, and contractility. Often researchers revert to using EF as a measure of systolic function or contractility. Other commonly misused parameters include SV, MAP, CO, and FS (Eqs 2–5) which all are dependent upon contractility, but also on preload, afterload, and heart rate, and thus, cannot be considered indices of contractility (Lipshultz et al., 1994). Contemporary standards in the field compel these measurements to be performed *in vivo* to enhance the reliability, relevance, and translational aspects.

Thus, researchers should explore nonterminal procedures that can properly evaluate of all aspects of heart function. Parameters of cardiovascular performance, heart performance, systolic function, diastolic function, and contractility with their proper corresponding accurate measurement are listed in Table 1. Precise measurements of all tiers individually are needed to fully evaluate cardiovascular function. Technology has advanced to tools in which this can now be performed by using speckle tracking echocardiography. In fact, speckle tracking is already widely used in the clinics. With technological advancement, these tools are now available for measurements in the most commonly used animal model in research: the mouse. Thus, researchers should recognize that EF is an antiquated measurement that poorly assesses heart function because of its reliance on physiological parameters and instead explore speckle tracking echocardiography.

## 3 Speckle tracking echocardiography

For basic research application, echocardiography has the advantage of longitudinal measurements for changes in heart function over time, rather than being the endpoint of an experiment. Traditional methods of echocardiography acquisition are often performed in M-mode, which traces the movement of the heart walls that occurs on a line drawn through the left ventricle. Besides misusing parameters obtained, this technique also introduces many sources of error and variation between measurements (i.e., the angle of the line drawn, the position of the probe in acquisition, the consistency of the placement by the user between animals, etc.) as well as sources of variation in analysis (Chukwu et al., 2008). Despite being well utilized in clinics, echocardiography has only recently been utilized to the full extent of its capabilities in basic research labs. STE has quantitative capabilities to truly measure systolic and diastolic function and contractility (Marwick et al., 2009; Morris et al., 2012; Oleynikov et al., 2018). STE uses B-mode, which is often the same imaging modality as an M-mode, but records a video clip of the entire heart wall rather than movement along one line. Therefore, as an average measurement, STE provides data points along the entire heart wall rather than only two from M-mode, which increases its accuracy as an indicator of heart function (Wang et al., 2018).

**FIGURE 2:**
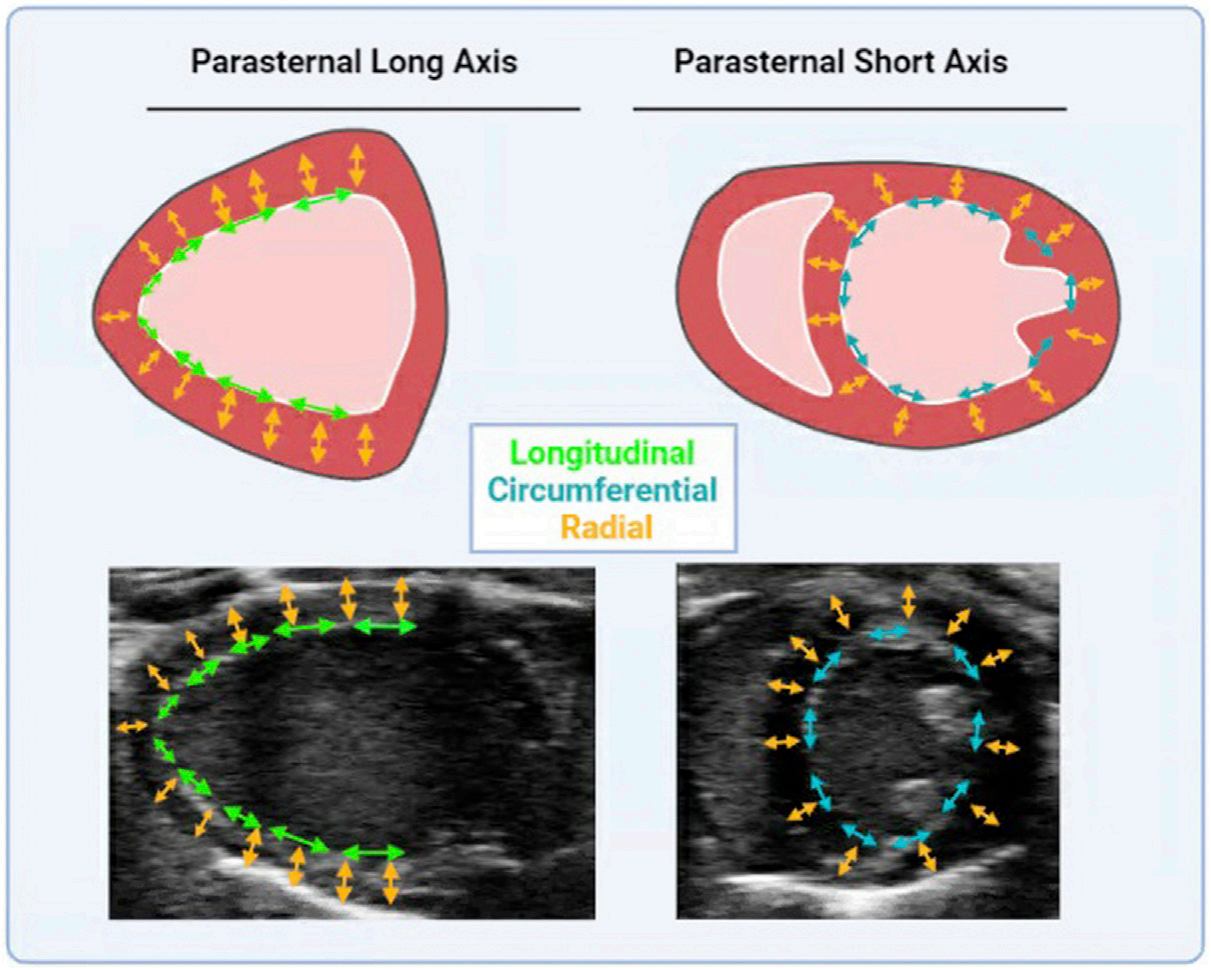
Directional vectors of Cardiac Strain. Cardiac strain is measured in parasternal long axis and short axis. Long axis cine loops provide long axis radial (in yellow arrows) and longitudinal strain (in green arrows). Short axis cine loops provide short axis radial (in yellow arrows) and circumferential strain (in red arrows). Cartoon and anatomical views are provided.

STE has only in recent years infiltrated basic cardiovascular sciences as a means of measuring heart function in research models (de Lucia et al., 2019). STE analysis performed on B-mode images traces the naturally occurring acoustic markers in a cine loop of the heart. Calculated from STE is displacement, velocity, strain, and strain rate in radial, longitudinal, and circumferential axes (see Figure 2). The axes of cardiac strain are dependent upon directionalities of the vector in the wall movement (Eq. 6). Cardiac strain delineates the deformation of the myocardium from diastole to systole, thus indicating systolic function. Strain rate is the derivative of strain with respect to time, and thus has distinct systolic and diastolic peaks (Eq. 7). Relative to EF, cardiac strain exhibits less load dependence, and strain rate is the least load dependent measurement of cardiac function thus far. Systolic strain is preload and heart rate dependent while systolic and diastolic strain rate have little preload dependence and no heart rate dependence (Sutherland et al., 2004; Hoit, 2011; Salvo et al., 2015). Comparisons between longitudinal, circumferential, and radial strain (and their corresponding strain rates) with EF and pressure-volume derived data reveal that strain and strain rate were weakly associated with load (arterial elastance and EDV). Strain and strain rate are more strongly associated with chamber elastance and contractility as compared to EF, which has a modest correlation with arterial elastance yet no correlation with chamber elastance (Zhang et al., 2014). Other studies have correlated strain and strain rate with pressure-volume loop- derived contractility indices, ESPVR (or End Systolic Elastance- Ees) and diastolic indices, EDPVR, specifically longitudinal strain and strain rate as well as circumferential strain and strain rate (Park et al., 2016). Strain has been shown to be less load dependent than EF, as well as have correlations with previously established indices of load-independent function (Zhang et al., 2014). Thus, unlike the variable effects of Bowditch effect, Starling’s Law and sympathetic stimulation on EF (dependent on which effect is larger- the increase in EDV or SV), the Bowditch effect and sympathetic stimulation will increase strain, while there will be little effect of Starling’s Law since strain measurements are virtually load-independent (Boettler et al., 2005; Ferferieva et al., 2013). Thus, STE has numerous benefits, with the major benefit being that a truer measurement of the heart performance, systolic function, diastolic function, and contractility. This method gives researchers the ability to fully characterize the heart without performing a fatal procedure and thus introduces the possibility for longitudinal studies. Contrary to all other methods of measuring cardiac function, STE also has the least user-dependent variability for image acquisition (Muraru D et al., 2018).

Monitoring and maintaining a close to physiological heart rate during measurements of heart function is incredibly important for accurate recordings (Lindsey et al., 2018). Variability in heart rate results in inconsistent measurements and difficulty comparing experimental variables. Additionally, an increased heart rate changes functional parameters (i.e., heart rate positively correlates with SV) (Lindsey et al., 2018). The resting physiological heart rate of a mouse averages around 600 BPM and frame rates on most current echocardiography equipment struggle to meet frame rates to obtain accurate measurements. A frame rate of >100 fps is required for speckle tracking analysis which improves with increasing frame rate (D'Hooge et al., 2000). Frame rates can be improved by reducing image width which decreases scan size and improves tracing (Voigt et al., 2015). Additionally, recent improvements in technology in the basic sciences field (i.e., VevoF2, Visual Sonics) allows for frame rate acquisition of up to 400 fps, making it easier to obtain and analyze speckle tracking echocardiography at higher heart rates.

STE and its subsequent measurements also have the distinct advantage of deciphering early onset of disease, specifically in certain axes of strain. Circumferential strain and strain rate have been shown to be sensitive enough to detect early onset of disease. Strain and strain rate decrease earlier than EF, and thus can be used as earlier indicators of onset of disease. Circumferential strain has also been indicated as an independent predictor of negative outcomes and ventricular remodeling after myocardial infarction (Hung et al., 2010). Longitudinal strain also exhibits similar sensitivity to early onset of disease and is an indicator of negative outcomes after MI. Thus another major advantages of STE in both humans and animal models is the ability to decipher early onset of disease (Pastore et al., 2021).

STE can also elucidate regional functional measurements (Pastore et al., 2021). Regional data is not only unavailable through every other method of analysis, but can provide quantification of models of heart disease, such as myocardial infarction (i.e., infarct size) (Munk et al., 2010). For example, global longitudinal strain has been shown to be more effective at quantifying infarct size compared to standard echocardiographic indices, such as EF and end systolic volume (Munk et al., 2010). STE also provides indices of diastolic function from one data acquisition and analysis, compared to the need for multiple methods of measurement such as Power Doppler and M-mode. STE can measure diastolic strain rate, which has been shown to be advantageous over myocardial velocity and blood flow velocity for assessment of diastolic function (Rivas-Gotz et al., 2003; Wang et al., 2007). Additionally, analysis of diastolic function through STE removes Doppler-associated angulation errors and tethering artifacts of other diastolic measurement techniques (Choudhury A et al., 2017).

In summary, STE is the superior method of *in vivo* analysis of the tiers of heart performance, providing indices that measure not only heart structure, but cardiovascular performance and heart performance. Most importantly, STE is a nonterminal procedure that can provide indices of contractility not previously been accessible to basic research *via* echocardiography.

## 4 Conclusion

The purpose of the heart is to pump blood to meet the metabolic demands of the body. In order to meet these demands, the heart needs healthy components of all aspects of function, including proper systolic and diastolic function and contractility. Proper evaluation of all the tiers are essential for the determining mechanism of action, the development, and progression of heart disease. A critical hallmark and determining factor of heart disease is a blunted contractility (Houser and Margulies, 2003), which significantly contributes to systolic function, heart performance, and cardiovascular performance. Thus, elucidating all contributing factors to heart function (i.e., systolic and diastolic function and contractility) is significant to understand how well the heart is performing, mechanistic studies, and the possibility to develop heart disease in animal models. STE is an accurate and reliable method to measure all facets of cardiac performance with one all-encompassing measurement. Most importantly, STE is a noninvasive and nonterminal procedure that can be repeated at multiple time points to provide indices of contractility through longitudinal studies.

## 5 Equations

$$\mathrm{Ejection}\;\mathrm{Fraction}\left(EF\right)=\frac{EDV-ESV}{EDV}*100\%$$ (1)

$$\mathrm{Mean}\;\mathrm{Arterial}\;\mathrm{Pressure}=CO*\mathrm{Total}\;\mathrm{Peripheral}\;\mathrm{Resistance}=\frac{2}{3}DBP+\frac{1}{3}SBP$$ (2)

Stroke Volume (SV)=EDV−ESV (3)

Cardiac Output(CO)=SV*Heart Rate (4)

$$\mathrm{Fractional}\;\mathrm{Shortening}\left(FS\right)=\frac{\left(LVIDd-LVIDs\right)}{LVIDd}*100\%$$ (5)

$$\mathrm{Cardiac}\;\mathrm{Strain}=\frac{L\left(t\right)-L\left(t_{0}\right)}{L\left(t_{0}\right)}*100\%$$ (6)

$$\mathrm{Cardiac}\;\mathrm{Strain}\;\mathrm{Rate}=\frac{∆\frac{L\left(t\right)-L\left(t_{0}\right)}{L\left(t_{0}\right)}}{∆t}$$ (7)
